# Pathogen-specific risk of chronic gastrointestinal disorders following bacterial causes of foodborne illness

**DOI:** 10.1186/1471-230X-13-46

**Published:** 2013-03-08

**Authors:** Chad K Porter, Daniel Choi, Brooks Cash, Mark Pimentel, Joseph Murray, Larissa May, Mark S Riddle

**Affiliations:** 1 Enteric Diseases Department, Naval Medical Research Center, Silver Spring, MD, USA; 2 George Washington University, Washington, DC, USA; 3 Walter Reed National Military Medical Center, Bethesda, MD, USA; 4 Cedars-Sinai Medical Center, Los Angeles, CA, USA; 5 Mayo Clinic, Rochester, MN, USA

## Abstract

**Background:**

The US CDC estimates over 2 million foodborne illnesses are annually caused by 4 major enteropathogens: non-typhoid *Salmonella* spp., *Campylobacter* spp., *Shigella* spp. and *Yersinia enterocoltica*. While data suggest a number of costly and morbid chronic sequelae associated with these infections, pathogen-specific risk estimates are lacking. We utilized a US Department of Defense medical encounter database to evaluate the risk of several gastrointestinal disorders following select foodborne infections.

**Methods:**

We identified subjects with acute gastroenteritis between 1998 to 2009 attributed to *Salmonella* (nontyphoidal) spp., *Shigella* spp., *Campylobacter* spp. or *Yersinia enterocolitica* and matched each with up to 4 unexposed subjects. Medical history was analyzed for the duration of military service time (or a minimum of 1 year) to assess for incident chronic gastrointestinal disorders. Relative risks were calculated using modified Poisson regression while controlling for the effect of covariates.

**Results:**

A total of 1,753 pathogen-specific gastroenteritis cases (*Campylobacter*: 738, *Salmonella*: 624, *Shigella*: 376, *Yersinia*: 17) were identified and followed for a median of 3.8 years. The incidence (per 100,000 person-years) of PI sequelae among exposed was as follows: irritable bowel syndrome (IBS), 3.0; dyspepsia, 1.8; constipation, 3.9; gastroesophageal reflux disease (GERD), 9.7. In multivariate analyses, we found pathogen-specific increased risk of IBS, dyspepsia, constipation and GERD.

**Conclusions:**

These data confirm previous studies demonstrating risk of chronic gastrointestinal sequelae following bacterial enteric infections and highlight additional preventable burden of disease which may inform better food security policies and practices, and prompt further research into pathogenic mechanisms.

## Background

Infectious gastroenteritis (IGE) is caused by a myriad of viruses, bacteria and parasites. Approximately 47.8 million foodborne-related illnesses occur annually in the United States, costing upwards of $150 million to the healthcare economy [1,2]. The four major bacterial enteropathogens responsible for morbidity and mortality include non-typhoidal *Salmonella*, *Campylobacter*, *Shigella*, and *Yersinia enterocolitica*[2]. In addition to the significant burden of the acute illness associated with these infections, recent evidence suggests that these pathogens are linked with chronic health sequelae, including functional gastrointestinal disorders (FGD) such as irritable bowel syndrome (IBS), functional constipation, and functional dyspepsia [3-8]. As a heterogeneous complex of disorders, FGD account for approximately 50% of all visits to gastroenterology practices and result in significant morbidity in the affected patient [9,10]. Additionally, these disorders result in significant financial burden, with annual costs per case ranging from $1,000-$5000 in the US [11-16]. Furthermore, such infections have been associated with inflammatory bowel disease [17-19], celiac disease [20,21], and functional dyspepsia [3,22-24] which share symptom overlap with gastroesophageal reflux disease (GERD) [25].

While numerous studies have reported on post-infectious FGD (PI-FGD), studies of pathogen-specific associations are limited. Such data are important, considering the need to account for food-borne illness risks in order to develop mitigating policies and practices, as well as to understand pathogenic mechanisms of PI-FGD. Thus, we sought to evaluate the risk of select chronic gastrointestinal consequences following illness attributed to non-typhoid *Salmonella*, *Campylobacter*, *Shigella*, and *Yersinia enterocolitica* in a young, predominately healthy subset of the US population.

## Methods

This was a retrospective cohort study in which the medical records of subjects diagnosed with non-typhoidal *Salmonella* spp*., Shigella* spp*., Campylobacter* spp., or *Yersinia enterocolitica* were assessed with the onset of FGD and GERD. Data were obtained from the Armed Forces Health Surveillance Center (AFHSC), which oversees the Defense Department Medical Surveillance System (DMSS), the main data repository for all medical encounters of active duty US military personnel.

Medical encounter history on all subjects with an inpatient or outpatient visit in which one of the following (International Classification of Diseases, 9^th^ Revision) ICD-9 codes were assigned was obtained: *Salmonella* (003.0 and 003.9), *Shigella* (004*), Campylobacter* (008.43) and *Yersinia enterocolitica* (008.44). Subjects with one of the foodborne illnesses identified above were matched by age, gender, number of deployments, medical treatment facility, encounter type (inpatient, outpatient) and time, to up to 4 subjects without a documented gastrointestinal infection. Baseline diagnoses of unexposed subjects included: acute respiratory infections, pneumonia and influenza, infections of skin and subcutaneous tissue, dislocations, sprains and strains for joints and adjacent muscles, superficial injury, burns, and fracture of the upper or lower limb.

Incident FGD and GERD were similarly identified using medical encounters with specific ICD-9 codes in any diagnostic position as follows: constipation (656.0), irritable bowel syndrome (564.1, 306.4), dyspepsia (536.8), and GERD (530.81). Other covariates obtained included race, military rank, socioeconomic factors (level of education, marital status), and Axis I and Axis II psychological conditions. All subjects with antecedent FGD or GERD diagnoses were excluded.

A modified Poisson regression analysis was used for data analysis with a robust sandwich estimator for variance [26]. Associations were initially explored by univariate methods. Multivariate models were utilized to assess the association between foodborne illness and FGD while controlling for important covariates. Initial models were developed utilizing all exposed subjects using a backwards elimination approach with an alpha of 0.15. Those models were then applied to pathogen-specific exposures. Two-tailed significance was evaluated using an alpha of 0.05. All analyses were performed using SAS 8.2 (Cary, NC).

The study protocol was approved by the Naval Medical Research Center Institutional Review Board (study protocol NMRC.2011.0003) in compliance with all applicable Federal regulations governing the protection of human subjects.

## Results

We identified 1,753 active duty US military personnel diagnosed with one of the four pathogens of interest (*Campylobacter*: 738, *Salmonella*: 624, *Shigella*: 376, *Y. enterocolitica*: 17) between 1998 and 2009 (Table 1). A total of 6,765 subjects were included in the referent cohort with baseline diagnoses that included respiratory or skin infections, sprains, strains, dislocations and fractures, superficial injuries and burns. In general, the demographics of the study population were representative of the active duty military population in terms of race (68.5% Caucasian), education (62% with only a high school education or equivalent), branch of service (31.1% Navy, 27.3% Army, 26.7% Air Force, 10.1% Marines, and 4.7% Coast Guard), rank (81.7% enlisted), and marital status (69% married). However, a higher proportion of Navy personnel were included in the referent cohort than in subjects with documented bacterial gastroenteritis (34.5% and 17.8%, respectively; p < 0.001). The median follow-up duration was 3.8 (interquartile range [IQR]: 2.1, 6.7) years, totaling 38,844 person-years of observation. A greater proportion of exposed Army personnel had bacterial IGE during the study period (45.0% for those exposed compared to 2.4 – 27.7% for the other branches of service, respectively; p < 0.001).

**Table 1:** Demographics of a cohort of U.S. military service members exposed with bacterial gastroenteritis infections and a matched (age, gender, deployment, baseline medical encounter) cohort of subjects without gastroenteritis

	**Exposed**					**Unexposed**
	**Any bacillary diarrhea**	***Campylobacter *spp.**	**Non-typhoid *Salmonella *spp.**	***Shigella *spp.**	***Yersinia enterocolitica***	
N	1753	738	624	376	17	6765
Median age (IQR)	28 (23,34)	29 (24,36)	26 (22,32)	29 (25,35)	27 (23,33)	28 (23,34)
Race [n (%)]						
White, Non-Hispanic	1264 (72.1)	584 (79.1)	424 (68.0)	242 (64.4)	16 (95.1)	4570 (67.6)
African-American	275 (15.7)	58 (7.9)	119 (19.1)	97 (25.8)	1 (5.9)	1291 (19.1)
Other	214 (12.2)	96 (13.0)	81 (13.0)	37 (9.8)	0 (0.0)	904 (13.4)
Sex [n (%)]						
Male	1419 (81.0)	624 (84.6)	496 (79.5)	287 (76.3)	14 (82.4)	5506 (81.4)
Female	334 (19.1)	114 (15.5)	128 (20.5)	89 (23.7)	3 (17.7)	1259 (18.6)
Branch of Service [n (%)]						
Army	789 (45.0)	378 (51.2)	252 (40.4)	153 (40.7)	8 (47.1)	1540 (22.8)
Marine	124 (7.1)	41 (5.6)	58 (9.3)	23 (6.1)	2 (11.8)	739 (10.9)
Navy	312 (17.8)	106 (14.4)	136 (21.8)	68 (18.1)	2 (11.8)	2336 (34.5)
Air Force	486 (27.7)	204 (27.6)	152 (24.4)	125 (33.2)	5 (29.4)	1790 (26.5)
Coast Guard	42 (2.4)	9 (1.2)	26 (4.2)	7 (1.9)	0 (0.0)	360 (5.3)
Education [n (%)]						
No High School	12 (0.7)	2 (0.3)	5 (0.8)	5 (1.3)	0 (0.0)	36 (0.5)
High School	961 (54.9)	357 (48.4)	378 (60.6)	213 (56.7)	14 (82.4)	4272 (63.2)
Less than 4 yrs of College	228 (13.0)	99 (13.4)	79 (12.7)	51 (13.6)	0 (0.0)	731 (10.8)
Bachelor’s Degree	257 (14.7)	121 (16.4)	84 (13.5)	51 (13.5)	1 (5.9)	819 (12.1)
Graduate Degree	234 (13.4)	135 (18.3)	54 (8.7)	44 (11.7)	1 (5.9)	628 (9.3)
Unknown	61 (3.5)	24 (3.3)	24 (3.9)	12 (3.2)	1 (5.9)	279 (4.1)
Marital Status [n (%)]						
Single, Never Married	347 (19.8)	129 (17.5)	153 (24.5)	61 (16.2)	5 (29.4)	1892 (28.0)
Married	1278 (72.9)	560 (75.9)	429 (68.8)	281 (74.7)	9 (52.9)	4564 (67.5)
Other	126 (7.2)	49 (6.6)	41 (6.6)	33 (8.8)	3 (17.7)	309 (4.6)
Unknown	2 (0.1)	0 (0.0)	1 (0.2)	1 (0.3)	0 (0.0)	0 (0.0)

The most common outcome was GERD (n = 943) followed by functional constipation (n = 373), IBS (n = 203) and dyspepsia (n = 190). The incidence (per 100,000 person-years) of each of these outcomes stratified by exposure is shown in Table 2. We saw a consistently higher incidence of FGD and GERD for those with prior IGE compared to the referent cohort. The strongest association was for IBS with *Y. enterocolitica* demonstrating the greatest relative risk (aRR: 13.1; 95% confidence interval [CI]: 4.4, 39.4). Functional dyspepsia showed only a weak association with bacillary diarrhea (Table 3).

**Table 2:** **Incidence (95**% **confidence interval) of chronic health outcomes per 100,000 person-years in a reference cohort or following documented bacillary diarrhea attributable to *Salmonella*, *Campylobacter*, *Shigella *or *Yersinia *among active duty U.S. military personnel from 1998 to 2009**

	**Any bacterial IGE**	***Salmonella***	***Campylobacter***	***Shigella***	***Yersinia***	**Unexposed**
**Irritable bowel syndrome**	3.0 (2.5, 3.7)	3.1 (2.2, 4.4)	2.9 (2.1, 4.0)	2.6 (1.6, 4.1)	13.2 (4.4, 39.7)	1.0 (0.9, 1.2)
**Functional Constipation**	3.9 (3.2, 4.6)	3.4 (2.5, 4.7)	4.0 (3.0, 5.2)	4.2 (2.9, 6.1)	8.5 (2.6, 28.0)	2.3 (2.1, 2.6)
**Functional Dyspepsia**	1.8 (1.3, 2.3)	1.4 (0.9, 2.3)	2.6 (1.9, 3.7)	0.8 (0.3, 1.8)	--	1.2 (1.0, 1.5)
**GERD**	9.7 (8.6, 10.9)	8.4 (6.8, 10.4)	10.9 (9.2, 12.9)	9.5 (7.4, 12.2)	13.9 (4.8, 40.5)	6.2 (5.8, 6.7)

**Table 3:** **Unadjusted and adjusted**^**1 **^**relative risk (with 95**% **confidence intervals) of chronic health outcomes following documented bacillary diarrhea attributable to *Salmonella*, *Campylobacter*, *Shigella*, and *Yersinia *among active duty U.S. military personnel from 1998 to 2009**

		**All bacterial IGE**	***Salmonella***	***Campylobacter***	***Shigella***	***Yersinia***
**Unadjusted effect estimates**	**IBS**	2.9 (2.2, 3.8)	3.0 (2.0, 4.4)	2.9 (2.0, 4.1)	2.5 (1.5, 4.2)	12.8 (4.2, 39.3)
	**Functional Dyspepsia**	1.4 (1.0, 2.0)	1.2 (0.7, 2.0)	2.1 (1.5, 3.1)	0.6 (0.3, 1.5)	--
	**Functional Constipation**	1.7 (1.3, 2.1)	1.5 (1.1,2.1)	1.7 (1.3, 2.3)	1.8 (1.2, 2.6)	3.6 (1.1, 12.1)
	**GERD**	1.6 (1.4, 1.8)	1.4 (1.1, 1.7)	1.8 (1.5, 2.1)	1.5 (1.2, 2.0)	2.2 (0.8, 6.5)
**Adjusted effect estimates**^**1**^	**IBS**	2.7 (2.1, 3.6)	2.8 (1.9, 4.2)	2.8 (1.9, 4.1)	2.3 (1.4, 3.9)	13.1 (4.4, 39.4)
	**Functional Dyspepsia**	1.3 (1.0, 1.9)	1.1 (0.6, 1.9)	2.0 (1.3, 3.0)	0.6 (0.2, 1.3)	--
	**Functional Constipation**	1.6 (1.3, 2.0)	1.4 (1.0, 1.9)	1.8 (1.3, 2.5)	1.6 (1.1, 2.4)	4.4 (1.2, 15.4)
	**GERD**	1.6 (1.4, 1.8)	1.5 (1.2, 1.8)	1.7 (1.4, 2.1)	1.5 (1.1, 1.9)	2.3 (0.8, 6.9)

^1^ Effect estimates were adjusted for specific covariates as follows:

IBS: branch of military service, ethnicity, gender.

Dyspepsia: marital status, branch of military service, gender, age at initiation of surveillance.

Functional constipation: marital status, military rank, branch of military service, sex, age at initiation of surveillance.

GERD: marital status, military rank, gender.

**Figure 1:**
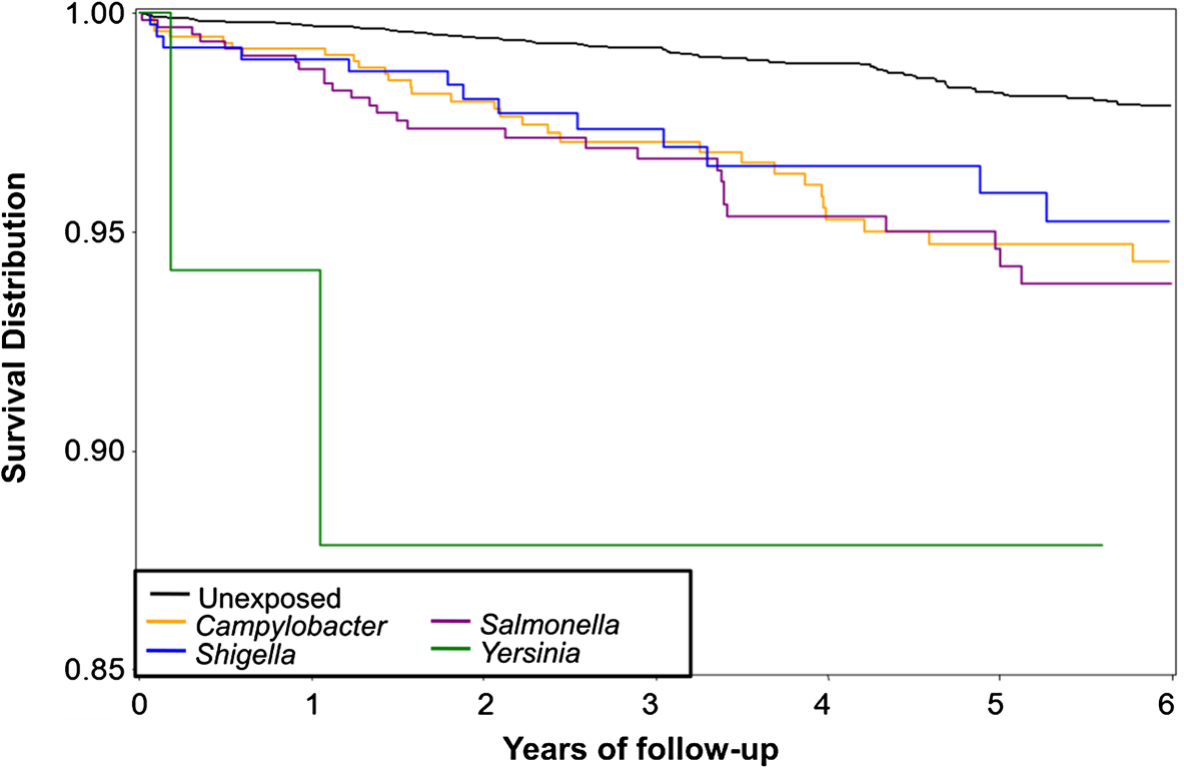
**Time to IBS onset following documented bacillary diarrhea attributable to *Salmonella*, *Campylobacter*, *Shigella*, and *Yersinia *among active duty U.S. military personnel from 1998 to 2009.**

We also noted variability in effect estimates across pathogen type. The highest adjusted relative risk for IBS was associated with prior *Y. enterocolitica* infection (aRR: 13.1; 95% CI: 4.4, 39.4); however, the number of cases was small. *Y. enterocolitica* also conferred significant risk for functional constipation (aRR: 4.4; 95% CI: 1.2, 15.4). The greatest pathogen variability occurred for the diagnosis of dyspepsia, ranging from an adjusted relative risk of 0.6 for *Shigella* (95% CI: 0.2, 1.3) to 2.0 for *Campylobacter* (95% CI: 1.3, 3.0). In addition, there was a more rapid rate of meeting the case definition, specifically for IBS, across all exposure types (Figure 1).

Across the pathogen-specific exposures, GERD diagnoses commonly overlapped functional GI disorders (Table 4). This overlap was more common amongst the exposed subjects, where 48.3% of IBS-diagnosed individuals also received a diagnosis of GERD during their follow-up compared to only 34.2% in unexposed subjects (p = 0.04). Similarly, amongst exposed subjects with constipation, 36.5% were also diagnosed with GERD compared to only 24.8% who were unexposed (p = 0.02).

**Table 4:** **Overlap of diagnosed outcomes following documented bacillary diarrhea attributable to *Salmonella*, *Campylobacter*, *Shigella*cpa and *Yersinia *and an unexposed reference cohort**

**Cohort**	**Outcome**	**IBS**	**Functional constipation**	**GERD**	**Functional dyspepsia**
Exposed	IBS	100%	22.5%	48.3%	11.2%
	Functional Constipation	--	100%	36.5%	10.4%
	GERD	--	--	100%	11.6%
Unexposed	IBS	100%	21.1%	34.2%	10.5%
	Functional Constipation	--	100%	24.8%	7.4%
	GERD	--	--	100%	11.5%

## Discussion

Our results support an accumulating body of epidemiological evidence for post bacterial gastroenteritis risk for FGD, including IBS, functional dyspepsia, and constipation, as well as GERD, which to our knowledge is an unrecognized putative sequel of IGE. Similar to prior studies, our results show a significantly increased risk of FGD among those with antecedent bacterial IGE [3,23,24,27,28]. Additionally, we observed variability in effect estimates across pathogens. Specifically, we found a greater risk of FGD in those with prior *Y. enterocolitica* infection than what was observed with other bacterial enteropathogens. Prior studies have documented a history of *Y. enterocolitica* infection in adults and children with persistent abdominal pain and other GI complaints [29,30]. In a 2004 follow-up study of laboratory confirmed enteric infections, 9.3% of survey respondents reported persistent GI symptoms following recovery from acute illness [31]. This proportion was higher for *Y. enterocolitica* (25%) than for *Campylobacter* (8.6%), *Salmonella* (8.0%), or *Shigella* (6.2%), although the overall response rate was low.

*Y. enterocolitica* is also known to cause granulomatous disease and thus may be a stronger trigger for subsequent immune dysregulation [32]. The association between FGD and antecedent IGE has been reported in several independent studies and systematic reviews [27,33]. In contrast to the published literature, the magnitude of increased risk reported here was lower than prior studies, possibly due to differences in study design and exposure and outcome ascertainment. However, this is the first study to report variable pathogen-specific risks across four common bacterial enteropathogens in a single population. Variability in the site of infection, method of invasion, and induction of the host immune responses due to these four enteropathogens are all possible contributors to the observed variability [34].

Studies of non-IBS PI-FGD are limited, although there are increasing reports on the risk of functional dyspepsia following acute enteric infection [3,5,23]. We recently reported an increased risk of FGD sequelae following all-cause IGE not limited to specific bacterial etiologies [6,7]. While the effect estimates here are lower than previously reported, they are in the same direction, and variability may be due to the pathogens studied, sample population, or other methodological differences. Despite the accumulating evidence linking IGE with functional dyspepsia, studies on the post-infectious risk of GERD are lacking. Thus, our finding of an association of enteric bacterial pathogen exposure with an incident diagnosis of GERD is, to our knowledge, a novel finding requiring further exploration. A complicating factor here is the diagnosis of GERD which can be distinguished by a variety of modalities including therapeutic trial, endoscopy, esophageal acid and motility testing, and gastric emptying studies [35]. Our outcome based on medical encounter ICD9-CM did not allow for verification of cases based on differential diagnostic criteria. Interestingly, our effect estimates of functional dyspepsia and GERD are similar, suggesting diagnostic misclassification of upper gastrointestinal dysfunction, or similar pathoetiological mechanisms resulting in these gastroduodenal disorders.

Also problematic in evaluating causation is the possibility that individuals may have used antacid medications (prescription or over-the-counter) for symptoms preceding their GERD diagnosis, which could have increased susceptibility to IGE and confounded the association between IGE and GERD. Confirmatory studies utilizing better-defined diagnostic criteria, database case-validation methods, and control of concomitant medications are needed.

We noted a significant amount of overlap in many of the outcomes; however, overlaps appeared to be more frequent among subjects with an antecedent bacillary diarrhea compared with those who were unexposed. Prior studies on post-infectious IBS following gastroenteritis attributed to non-typhoid *Salmonella* have pointed to an overlap with dyspepsia [23] and an increased risk of PI-dyspepsia has been reported following numerous IGE exposures [3,6-8,24,36]. While FGDs are often characterized by the Rome criteria [37], such classification may be insufficient at characterizing the true phenotypic classification and symptom complex seen in patients with PI-FGD. Future studies to better characterize the outcome of PI-FGD are needed to expand the phenotypic attributes of these functional outcomes following IGE.

A number of clinical features have previously been shown to be risk modifiers for PI-FGD including severity of disease, sex, use of antibiotics, psychological comorbidities, acute stress, and duration of illness [38-40]. The medical encounter data utilized precluded any clinical symptom-based severity of disease assessment, however we did not identify sex as an important covariate across the FGD models. Furthermore, we were also able to evaluate the potential influence of comorbid psychological conditions on both host susceptibility to enteric infection, as well as the potentiation of FGD after enteric infection. No association between diagnosed psychological disorders and FGD risk in this study was identified (data not shown), possibly due to the reliance on diagnosed mental health conditions instead of self-reported conditions. Several prior studies have identified an association between stressful events prior to infectious episodes and the subsequent risk of FGD [41,42]. However, we were unable to assess undiagnosed stressors or other psychosocial comorbidities prior to either exposure or outcome.

Interpretation of these results should be done with a full appreciation of potential biases inherent with medical encounter database studies, as well as the population under study. Active duty military personnel are younger and generally healthier than other adults, therefore the associations and effect estimates may not be generalizable to other populations. Secondly, because we relied on ICD-9 codes and reportable disease notification data rather than actual laboratory confirmation, we cannot assume that IGE was laboratory-confirmed in all cases, although presumably a pathogen-specific ICD-9 would not be coded without confirmation. Because our data set did not include pharmacy data or survey-based symptom assessment, we could not completely control for pre-existing functional symptoms, nor the use of proton pump inhibitors, antacids, or other medications, which may have confounded the observed associations with both exposure and outcomes [43]. An attempt was made to control for diagnosed medical conditions in which acid suppressive medication would be used (e.g. peptic ulcer disease, Barrett’s esophagus); however, future studies are needed to control for actual medication usage and pre-existing conditions. Similarly, the use of antibiotics for IGE treatment may have increased exposed subjects’ risk of FGDs; which, as previously described, may be confounded by subjects with more severe clinical illness [40].

## Conclusions

Despite the noted limitations, we have confirmed an increased risk of FGDs following infection with unique bacterial enteropathogens. Recent reports on the significant costs and morbidity associated with the acute disease attributable to foodborne illness [44,45], and the significant costs and decrements in health-related quality of life associated with the long-term health outcomes reported here [11,12,15,16,46], highlight the need for continued efforts to improve primary prevention strategies and to better understand the etiology underlying the differential pathogen-specific risks. While additional study is needed, these results raise the specter of the potential burden associated with these infections, and highlight the need for primary prevention strategies and optimized food safety policies in the US and globally.

## Consent

This study was conducted using a waiver of informed consent.

## Competing interests

The authors have no competing interest.

## Authors’ contributions

CKP and MSR conceived of the study and CKP, DC and MSR carried out all descriptive and statistical analyses and drafted and edited the manuscript. BC, MP, JM and LM participated in its design and coordination and helped to draft the manuscript. All authors read and approved the final manuscript.

## Authors’ information

Authors are employees of the U.S. Government and military service members. This work was prepared as part of official duties. Title 17 U.S.C. §105 provides that ‘Copyright protection under this title is not available for any work of the United States Government.’ Title 17 U.S.C. §101 defines a U.S. Government work as a work prepared by a military service member or employee of the U.S. Government as part of that person’s official duties.

## Pre-publication history

The pre-publication history for this paper can be accessed here:

http://www.biomedcentral.com/1471-230X/13/46/prepub

## Disclaimer

The views expressed in this article are those of the author and do not necessarily reflect the official policy or position of the Department of the Navy, Department of Defense, nor the U.S. Government. This is a US Government work. There are no restrictions on its use. There were no financial conflicts of interests among any of the authors. This study was conducted under support of the Military Infectious Disease Research Program.
